# Morphological adaptation for ectosymbiont maintenance and transmission during metamorphosis in *Lagria* beetles

**DOI:** 10.3389/fphys.2022.979200

**Published:** 2022-08-30

**Authors:** Rebekka S. Janke, Safira Moog, Benjamin Weiss, Martin Kaltenpoth, Laura V. Flórez

**Affiliations:** ^1^ Department of Evolutionary Ecology, Institute of Organismic and Molecular Evolution, Johannes Gutenberg University, Mainz, Germany; ^2^ Department of Insect Symbiosis, Max Planck Institute for Chemical Ecology, Jena, Germany; ^3^ Department of Plant and Environmental Sciences, Section for Organismal Biology, University of Copenhagen, Copenhagen, Denmark

**Keywords:** symbiotic organ, *Lagriinae*, *Burkholderia*, metamorphosis, symbiont transmission, ectosymbiont maintenance, morphological adaptation

## Abstract

The diversity and success of holometabolous insects is partly driven by metamorphosis, which allows for the exploitation of different niches and decouples growth and tissue differentiation from reproduction. Despite its benefits, metamorphosis comes with the cost of temporal vulnerability during pupation and challenges associated with tissue reorganizations. These rearrangements can also affect the presence, abundance, and localization of beneficial microbes in the host. However, how symbionts are maintained or translocated during metamorphosis and which adaptations are necessary from each partner during this process remains unknown for the vast majority of symbiotic systems. Here, we show that *Lagria* beetles circumvent the constraints of metamorphosis by maintaining defensive symbionts on the surface in specialized cuticular structures. The symbionts are present in both sexes throughout larval development and during the pupal phase, in line with a protective role during the beetle’s immature stages. By comparing symbiont titer and morphology of the cuticular structures between sexes using qPCR, fluorescence *in situ* hybridization, and micro-computed tomography, we found that the organs likely play an important role as a symbiont reservoir for transmission to female adults, since symbiont titers and structures are reduced in male pupae. Using symbiont-sized fluorescent beads, we demonstrate transfer from the region of the dorsal symbiont-housing organs to the opening of the reproductive tract of adult females, suggesting that symbiont relocation on the outer surface is possible, even without specialized symbiont adaptations or motility. Our results illustrate a strategy for holometabolous insects to cope with the challenge of symbiont maintenance during metamorphosis via an external route, circumventing problems associated with internal tissue reorganization. Thereby, *Lagria* beetles keep a tight relationship with their beneficial partners during growth and metamorphosis.

## Introduction

Microbial symbionts are widespread in nature and can be found in various body parts of animals, sometimes forming tight associations with their hosts (Buchner, 1953). Often, host morphological modifications for housing and guiding symbionts ensure the establishment and maintenance of mutualistic interactions (Salem et al., 2015; Hammer and Moran, 2019). In some animal-microbe symbioses, particular tissues, structures, or organs are likely adapted to favor specific microbial inhabitants (Douglas, 2020) like bacteriocytes in aphids (Braendle et al., 2003) and other sap-sucking insects (Baumann, 2005), or grain beetles (Hirota et al., 2017; Engl et al., 2018), light organs in squids (Belcaid et al., 2019), trophosomes in tubeworms (Bright and Giere, 2005; Southward et al., 2005), antennal reservoirs of solitary digger wasps (Kaltenpoth et al., 2005; Goettler et al., 2007, 2022), or midgut ceca in bean bugs (Ohbayashi et al., 2015).

In insects, symbiont locations are as diverse as their functional contributions to their hosts. Generally, localization correlates with—and likely constrains—symbiont function. For example, defensive microbes often occur as ectosymbionts or in proximity to the outer surface (Flórez et al., 2015). In contrast, symbionts involved in digestion or detoxification are commonly found within or around the gut (Engel and Moran, 2013), while nutrient-supplementing symbionts are either localized in gut-associated organs or inhabit bacteriomes (Salem and Kaltenpoth, 2022). Symbiont localization and/or function can however change under different circumstances. In reed beetles, symbiont translocations from intracellular to extracellular sites in the host occur along with changes in symbiont function (Reis, 1931). Alternatively, symbionts can be lost when the contributions are no longer needed, as observed for the tyrosine-supplementing symbionts in the cereal weevil and the sawtoothed grain beetle (Vigneron et al., 2014; Engl et al., 2020). In particular, this occurs in adult males of several insect taxa, since they are not involved in passing symbionts to the next generation (Buchner, 1953).

To enable successful symbiont transmission across generations, different mechanisms and adaptations have evolved to relocate symbionts. Generally, symbionts can be acquired and transferred through strict vertical transmission from parents to offspring, via horizontal acquisition every new generation, or by a mixed-mode combining both (Bright and Bulgheresi, 2010; Salem et al., 2015). Many obligate intracellular endosymbionts that provide essential functions for host survival are transmitted transovarially through the female germ line (Buchner, 1953; Szklarzewicz and Michalik, 2017). In contrast, transmission of extracellular symbionts can be more diverse and sometimes flexible, often occurring through mechanisms that are in play during or after egg-laying (Salem et al., 2015).

Guaranteeing the maintenance or re-colonization of symbionts is not only a challenge over generations but also throughout different life stages. Reorganization of tissues, including symbiotic organs, is usually drastic during complete metamorphosis, posing a challenge on symbiont transmission and maintenance (Hammer and Moran, 2019). A defined translocation route for symbionts when insects mature has been described in a few cases (Stoll et al., 2010; Maire et al., 2020), sometimes involving shifts from intra- to extra-cellularity as internal structures reorganize (Estes et al., 2009). While the persistence of symbionts in the host will depend on the successful translocation during metamorphosis, this issue has so far received much less attention than the transmission across generations.

Here, we focus on the impact of symbiont localization and transmission during metamorphosis on the symbiosis between two *Lagria* beetle species and their bacterial symbionts. The presence of putative symbiont-bearing structures in the adults of 83 species of this subfamily of darkling beetles (Lagriinae, Tenebrionidae) was reported based on morphological observations (Stammer, 1929), yet the symbiotic association with bacteria and the corresponding housing structures in the larvae have been described specifically in *Lagria hirta* (Flórez and Kaltenpoth, 2017) and *Lagria villosa* (Flórez and Kaltenpoth, 2017, Janke et al., in review). *L. villosa* and *L. hirta* house a community of symbionts throughout their life cycle, dominated by bacteria of the genus *Burkholderia* (Flórez and Kaltenpoth, 2017; Flórez et al., 2017; Janke et al., in review). Hereinafter, we refer to the *Burkholderia* symbionts as the symbionts or ectosymbionts, although other bacterial taxa are also present in the community (Flórez et al., 2017; Flórez and Kaltenpoth, 2017). While occasional environmental exchange occurs, the symbionts are predominantly transmitted vertically from female accessory glands onto the egg surface during oviposition, from where they colonize three unusual dorsal cuticular invaginations of the mesothorax, metathorax, and the first abdominal segment in both sexes of larvae (Stammer, 1929; Flórez and Kaltenpoth, 2017; Flórez et al., 2017; Janke et al., in review). *L. villosa* pupae maintain the symbionts in similar structures on the surface, from where they presumably translocate to accessory glands of the reproductive tract in females. By contrast, male adults lack the symbionts. In addition, some strains can be transferred from females to the environment or horizontally acquired during the larval stage (Wierz et al., 2021). In *L. villosa*, the ectosymbionts protect the eggs and larvae against fungal infestation by producing antibiotics (Flórez et al., 2017, 2018; Janke et al., in review). One symbiont strain (*Burkholderia* Lv-StB, henceforth “Lv-StB”) consistently produces the antifungal compound lagriamide and likely plays a pivotal role in defense (Flórez et al., 2018; Janke et al., in review). Although this strain lacks common genes for motility (Waterworth et al., 2020), it successfully colonizes the larval stage, persists in all life stages in high numbers, and dominates the microbial community in the beetle (Janke et al., in review). It is unclear yet when symbionts are lost in male *Lagria* beetles and if they might be ecologically relevant for defense in both sexes also during pupation (Janke et al., in review). In *L. hirta* it was hypothesized that the symbionts are transmitted to the female accessory glands externally via the molting fluid (Buchner, 1953), however, how symbiont transmission and maintenance is facilitated during metamorphosis, especially for the presumably immotile Lv-StB, is not yet known. Therefore, we explored the pupal stages of *L. villosa* and *L. hirta* to shed light on the symbiosis at this stage. We compared symbiont titers via qPCR, visualized symbiont localization and morphology of the symbiotic organs in the host using histological sections and fluorescence *in situ* hybridization (FISH) and micro-computed tomography (µCT), and simulated a potential transmission route of Lv-StB using fluorescent beads and microscopy. Thereby, we demonstrate that symbiont loss in males already begins in the pupal stage, which is accompanied by morphological changes of the symbiotic organs during metamorphosis. Furthermore, we show that symbiont transmission from female pupae to adults probably occurs externally via the host surface. These findings indicate that the ecological importance of the symbionts likely drove the evolution of specialized structures in the host to house and maintain the bacteria during metamorphosis.

## Materials and methods

### Insect collecting and rearing

*L. hirta* individuals were collected in Germany in 2020 (Supplementary Table S1) and reared in a terrarium (80 cm × 120 cm × 135 cm) consisting of a mesh cage and a plastic container filled with watered soil and live blackberry plants. The terrarium was maintained outside under trees and a canvas cover to protect it against heavy rain and to simulate semi-natural rearing conditions over the winter. The offspring of these individuals were collected in 2021.

*L. villosa* individuals were collected in Brazil in 2019 (Supplementary Table S1), reared in plastic containers in a climate chamber (16:8 L:D light regime at 26°C and 60% humidity), and were fed with leaves of lettuce, soybean, and kidney bean.

### Sex determination in *Lagria* pupae

The sex determination of *Lagria* pupae was done by a combination of approaches. In both species, sex can be roughly estimated by size, since females are often bigger and heavier than males (Supplementary Figure S1A). An accurate sex determination was possible in early pupae based on the caudal abdominal region and the morphology of the genital papillae in both *Lagria* species (Supplementary Figures S1B–D). For *L. hirta* pupae, the sex can also be determined based on the last antennal segment, which is notably longer in males (Supplementary Figure S1E).

### Determination of symbiont titers using DNA extraction and qPCR

*L. villosa* individuals were collected in the field or as offspring of field-collected females and preserved dry at −80°C or in ethanol at −20°C. Adult females were dissected to obtain the symbiont-bearing structures, whereas entire individuals were used for all other life stages. Samples were homogenized with liquid nitrogen, and DNA extraction was carried out using the Epicentre MasterPure Complete DNA and RNA Purification Kit or the innuPREP DNA/RNA Mini Kit (Analytik Jena) following the manufacturer’s instructions. To analyze symbiont titers across *L. villosa* life stages, qPCRs were carried out targeting the gyrase B gene of all *Burkholderia* strains using the primers Burk_gyrB_F (5′-CTC​GAG​AAG​CTG​CGC​TAT​CA-3′) and Burk_gyrB_R (5′-GCG​ATA​GAG​GAA​CGT​GAG​CA-3′). To determine differences in symbiont titers between sexes in *L. villosa* and *L. hirta* pupae, qPCRs were conducted targeting the 16S rRNA gene targeting all known *Burkholderia* symbiont strains of *Lagria* beetles, using the primers Burk16S_1_F (5′-GTT​GGC​CGA​TGG​CTG​ATT-3′) and Burk16S_1_R (5′-AAG​TGC​TTT​ACA​ACC​CGA​AGG-3′). qPCRs were carried out using the 5x HOT FIREPol EvaGreen HRM Mix EvaGreen (Solis BioDyne) on a RotorGene-Q cycler (Qiagen) in 10 µl reactions including 0.5 µl of each primer and 1 µ template DNA under the following conditions: Initial activation at 95°C for 15 min, denaturation at 95°C for 15 s, annealing at 62°C for 15 s and elongation at 72°C for 15 s for 50 cycles. Standard curves were created by amplifying the fragment, followed by purification and determination of the DNA concentration using a Qubit fluorometer (Thermo Fisher). A standard containing 1 ng/μl was generated and 1:10 serial dilutions down to 10^−8^ ng/μl were prepared. All standards and no-template controls were included in the qPCR run for absolute quantification.

### Morphology of the cuticular structures of female and male pupae (µCT)

For each *L. villosa* pupal stage (early, middle, and late), two female and two male individuals were prepared for micro-computed tomography (µCT) analysis. To this end, samples were fixed in 4% PFA in 80% ethanol at room temperature for 48 h and then washed twice with 80% ethanol for 1 h each time under agitation. After samples were dehydrated in denaturated ≥99.8% ethanol for 48 h, contrasting was performed in freshly prepared 1% resublimated iodine (Carl Roth, Karlsruhe, Germany) in pure methanol at room temperature for 24 h. Subsequently, the samples were washed three times for 1 h each using denaturated ≥99.8% ethanol and under shaking conditions, followed by three times in pure ethanol. Drying was performed in an automated EM CPD300 critical point dryer (Leica Microsystems, Wetzlar, Germany) at medium speed CO_2_ supply with a delay of 20 min, with 20 exchange cycles, followed by heating at medium speed and slow gas exhaust. The dry specimens were attached upright to an approximately 5 mm piece of fishing line using a UV-curable adhesive Fotoplast Gel (Dreve Otoplastik, Unna, Germany) and mounted on the specimen holder.

All X-ray scans were performed using a SkyScan 1,272 microtomograph (Bruker, Kontich, Belgium) with 360° rotation, 0.2° rotation steps, and a frame averaging of 4. The average source voltage and current were adjusted to 39–100 kV and 100–200 μA, respectively, to generate consistent signal attenuation of approximately 35%. The average pixel size was 5–6 µm. The NRecon software (Bruker, Kontich, Belgium) was used for reconstruction and ring artifact correction.

Image analysis was performed using Dragonfly 2020.2 [Object Research Systems (ORS) Inc., Montreal, Canada, 2020; software available at http://www.theobjects.com/dragonfly]. TIFF image stacks were imported with an X:Y: Z ratio of 1:1:1 and precisely aligned within the clipping box. The segmentation and reconstruction of symbiotic organs was performed manually. 2D Videos were created in Dragonfly with a speed of 40 FPS.

### Evaluation of symbiont presence, localization, and host morphology using fluorescence *in situ* hybridization

*Lagria* larvae and pupae were fixated in 4% formaldehyde for at least 3 days. Embedding, semithin sectioning, and FISH were performed as described previously (Weiss and Kaltenpoth, 2016). One pupal-adult exuvia was placed on a glass slide with double-sided adhesive tape and fixated with 70% ethanol. The Cy3-or Cy5-labeled Burk16S probe (5′-TGC​GGT​TAG​ACT​AGC​CAC​T-3′) was used to mark all *B. gladioli* strains and the Cy3-labeled EUB338 probe (5′-GCT​GCC​TCC​CGT​AGG​AGT-3′) was used for general eubacteria. DAPI (4′,6-diamidino-2-phenylindole) was used to label the host cell nuclei and as counterstaining. Images were taken on an AxioImager.Z2 fluorescence microscope (Zeiss, Jena, Germany). To determine symbiont presence and localization in female and male pupae, sagittal sections of early pupae were imaged and relevant locations or areas containing symbionts were analyzed, while the exuvia was imaged as a whole. To find out when morphological changes of the symbiotic organs occur during female and male development, sagittal sections of a first instar larva, two late last instar larvae before pupation, and two pupae were analyzed focusing on the morphology of the symbiotic organs.

### Simulation of immotile symbiont transmission from pupa to adult using fluorescent beads

To simulate symbiont transmission of immotile bacterial symbionts*,* five early *L. villosa* pupae were inoculated with fluorescent beads (Sigma Aldrich, Latex beads, amine-modified polystyrene, fluorescent red, 1.0 μm mean particle size). Pupae were kept in separate wells of a 24-well plate, which was prepared with moist vermiculite and sterile filter paper. Fluorescent beads were diluted in PBS to a final concentration of 10^6^ beads/µl and 5 µl were added to the dorsal thorax at the region of the symbiont-bearing organs. Two freshly emerged female adults and one male as a control were then dissected step-by-step, starting with removal of the elytra and wings until the reproductive organs were fully visible. In between each dissection step, dissection tools were washed and images were taken to determine the location of the fluorescently labeled beads. Images were acquired on an AxioImager.Z2 fluorescence microscope (Zeiss, Jena, Germany).

## Results

### Symbiont presence in *Lagria* pupae

**FIGURE 1:**
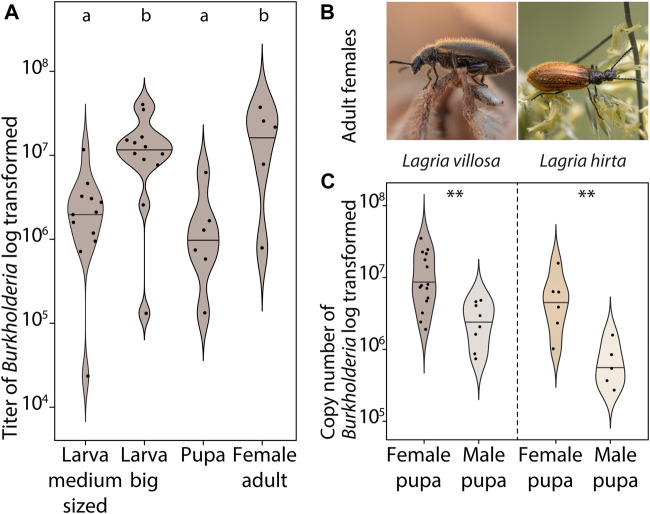
Symbiont abundance in male and female *Lagria* pupae. **(A)** Abundance of *B. gladioli* (gyrase B gene copies) of field-collected *L. villosa* larvae (sex is not known for medium sized and big larvae), pupae (sex unknown), and adult females. Different letters indicate significant differences between experimental treatments (Kruskal–Wallis test, χ2 = 14.3, df = 3, *p*-value = 0.0026, posthoc Dunn’s Test) **(B)** Photographs of *Lagria* females from the two evaluated species *L. villosa* (left, Brazil) and *L. hirta* (right, Germany). **(C)** Abundance of *B. gladioli* copies (16S rRNA gene of *B. gladioli*) of field-collected male and female pupae of *L. villosa* (left) and *L. hirta* (right). Asterisks indicate significant differences between females and males (Two Sample *t*-test, **p < 0.01).

Since there is evidence in *L. hirta* that both male and female larvae carry symbionts, but adult males lack them in both *Lagria* species (Stammer, 1929; Flórez and Kaltenpoth, 2017), we aimed to better understand the process and timing of symbiont loss. We initially compared symbiont titers of field-collected female adults, pupae, and larvae without separating immature stages by sex. In *L. villosa,* symbiont titers increase during larval development, but decrease in the pupal stage and finally reach the highest numbers in adult females (Figure 1A). To evaluate whether this lower symbiont titer in pupae was specific to a certain sex and if it is similar between the two *Lagria* species, we compared the symbiont titer of female and male *L. villosa* and *L. hirta* pupae (Figures 1B,C). Indeed, male pupae had significantly lower symbiont titers than females in both beetle species. Also, symbiont titers were generally lower in *L. hirta* than in *L. villosa*, possibly corresponding to the observed size differences between the species (Supplementary Figure S1A).

**FIGURE 2:**
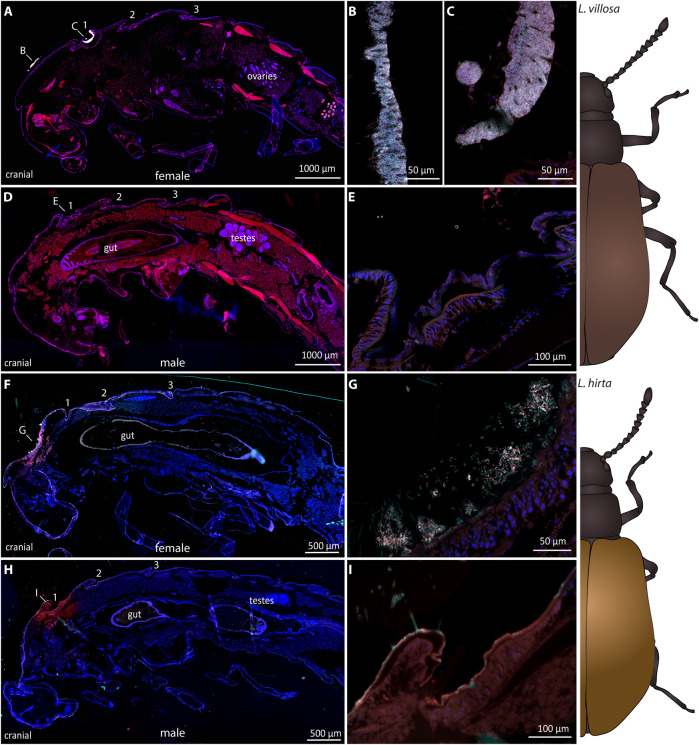
Sexual dimorphism of symbiotic organs and symbiont localization in *Lagria* pupae using FISH. FISH was carried out on semithin sagittal histological sections of *L. villosa* **(A–E)** and *L. hirta* **(F–I)** pupae. Symbiont cells are generally depicted in white, host cell nuclei in blue, and autofluorescence of the host tissue in red. **(A–E)** Symbionts were present in female *L. villosa* pupae **(A)** on the surface of the thorax between bristles **(B)** and within the first dorsal symbiont organ **(C)**, while symbionts were not detectable in male pupae **(D,E)**. **(F–I)** Symbionts were detected in *L. hirta* females **(F)** in a pit of the dorsal thorax between bristles **(G)** while they were not detectable in males **(H,I)**. *Burkholderia-specific* staining is shown in cyan, general eubacterial staining in red, and host cell nuclei in blue (DAPI). Overlapping signal is shown in white. Numbers indicate the location of symbiotic organs or cuticular structures in the respective position and all images show sagittal sections with the cranial end to the left.

To evaluate potential differences in the pupal symbiotic organs between the two sexes, we carried out FISH experiments on one female and one male *Lagria* pupa in each of the two species (Figure 2). In *L. villosa* pupae, females (Figure 2A) carried dense accumulations of symbionts mainly on the surface of the dorsal thorax (Figure 2B) and in the bigger first symbiotic organ (Figure 2C). In contrast, the first dorsal symbiotic organ is only vestigial in males (Figure 2D) and accumulations of symbionts were not observed (Figure 2E). In *L. hirta* pupae, the female (Figure 2F) showed an accumulation of symbionts in a pit of the dorsal thorax between bristles (Figure 2G), but not in the region of the first symbiont organ as observed in *L. villosa* pupae. In males, we did not detect the first organ nor any symbiont cells (Figures 2H,I). In summary, these results indicate that the symbiotic organs of male *Lagria* pupae degenerate during pupation and symbiont reservoirs in the respective parts are missing, which relates to a decline of symbionts during metamorphosis.

### Morphology of symbiotic organs in larvae and pupae

**FIGURE 3:**
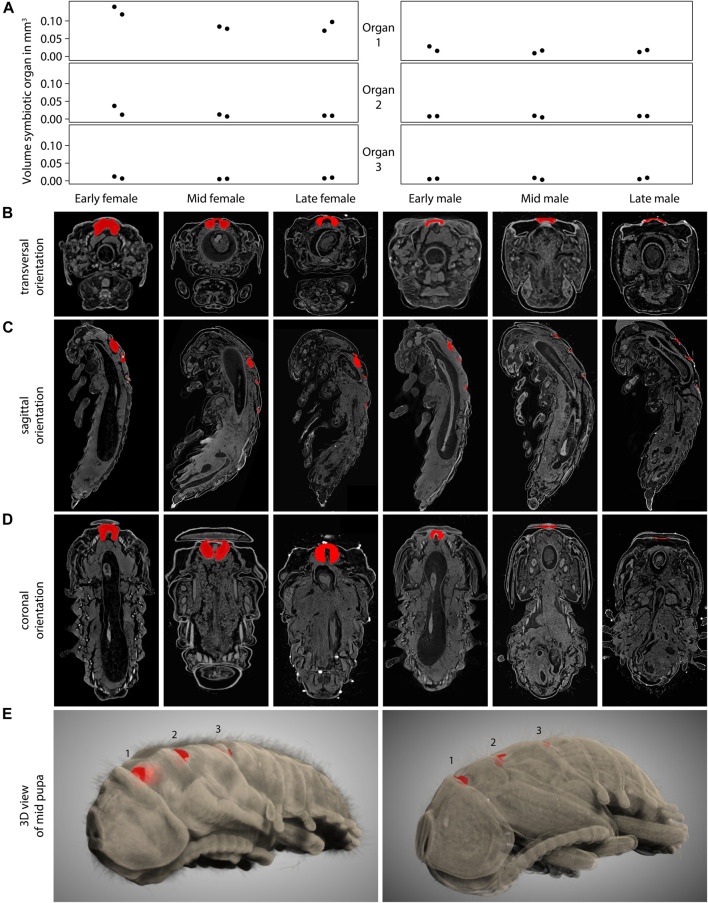
Symbiotic organs in female and male *L. villosa* across pupal development. **(A)** Volume of the symbiotic organs or cuticular structures measured from 3D µCT scans of *L. villosa* pupae of different sexes and stages. Female samples are shown on the left panels and males on the right. **(B–D)** Images show single sections in **(B)** transversal, **(C)** sagittal, and **(D)** coronal orientations through specimen (from left to right: early, mid and late female; early, mid, and late male pupa) corresponding to the columns in **(A)**. Cuticular structures are labeled in red and show the first organ in transversal and coronal sections, and all three structures in sagittal sections. **(E)** 3D representation of a female (left) and male (right) mid pupa showing the cuticular structures in red within the dorsal surface.

To identify if the observed decrease in symbiont titers, especially in male pupae, can be attributed to morphological differences in the cuticular symbiont-bearing structures, we used µCT imaging of female and male *L. villosa* pupae. We measured the total volume of female and male pupae and their cuticular structures at different time points (Figure 3A). The results indicate that although the total volume of pupae is not different across sexes and time points (approximately 100 mm^3^ for both sexes), the volume of the first structure differs between males and females. The bigger volume in females is in line with the observation of single sections via FISH (Figure 2). By looking at the whole 3D scan, we found that the first structure has a double-lobed morphology in females, while it is much less pronounced in males (Figure 3B). The lobes correspond to the bigger part of the structure, which are oriented towards the caudal side in a sagittal orientation (Figure 3C). A coronal view indicated that the first structure is similar across female time points but is progressively reduced during male pupal development (Figure 3D). 3D representations of the whole pupa (Figure 3E, Supplementary Videos S1, S2) also showed that generally, the first structure looks more like a specialized organ, while the second and third structure are visually similar to the cuticular folds between the other segments.

**FIGURE 4:**
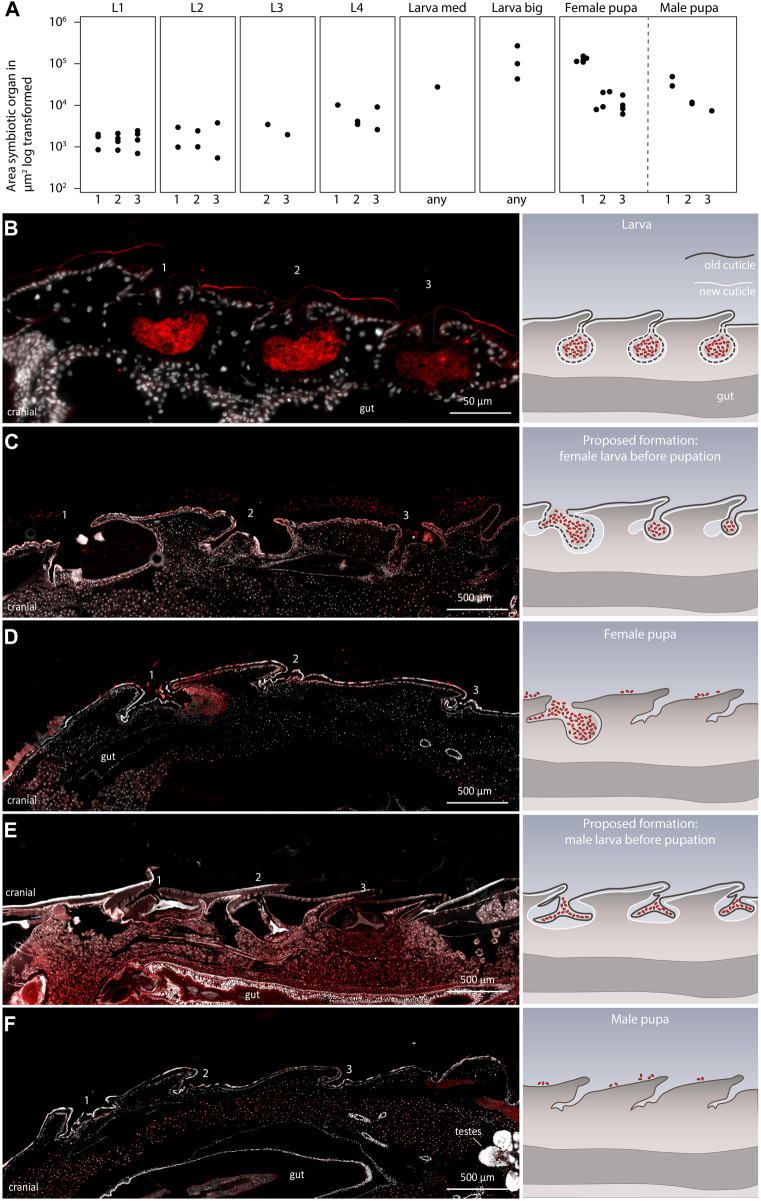
Development of the symbiotic organs in late *L. villosa* larvae and pupae. **(A)** Size of the symbiotic organs or cuticular structures measured in sagittal sections of *L. villosa* larvae and pupae. For standardization, sections of individuals which contained the opening canal of the symbiotic organ were chosen. However, variations in exact location, shape, and accuracy during sectioning possibly influenced the measured area. Numbers on the *x*-axis label refer to the first (1), second (2), third (3) symbiotic organ, or to an unknown (any) symbiotic organ or cuticular structure at the respective location. **(B–F)** The left panel shows relevant micrographs of histological sections covering the region of the three dorsal structures and the right panel shows illustrations representing the proposed morphological change of the organs from larvae to pupae in females and males, with the old cuticle in brown and the new cuticle in white. FISH was carried out on semithin sagittal histological sections of a *L. villosa* larva **(B)**, larvae right before pupation **(C,E)**, sex unknown but assumed), and female **(D)** and male **(F)** pupae. Numbers indicate the location of symbiotic organs or cuticular structures in the respective position and all images show cutouts of sagittal sections with the cranial end to the left.

During the identification of the sexual dimorphism in the symbiotic organs in *Lagria* pupae, we also observed that the morphology of the symbiotic organs generally changed from the larval to the pupal stage. *L. villosa* larvae have three equally sized dorsal symbiont organs (Figures 4A,B), which increase in size while the insect grows, as described for *L. hirta* (Stammer, 1929). However, female pupae have a bigger first organ, and the second and the third structures decrease in size (Figure 4A). Contrastingly, male pupae have three almost equally sized cuticle-lined structures, which are smaller in comparison to those in large larvae.

To identify at which time point this change in morphology occurs, we sectioned two late final instar larvae which were about to pupate and assessed the morphology of the symbiotic organs. Unfortunately, due to the lack of sex-specific morphological characters, we were not able to identify the sex of the larvae, but we found two different morphologies of symbiotic organs in the two specimens. In one specimen, we observed three organs that decreased in size from the first to the third organ (Figure 4C), which would be in line with an intermediate state leading to the morphology of an early female pupa (Figure 4D). In the other specimen, we found three similarly sized organs, which looked compressed and to some extent detached from the developing new cuticle (Figure 4E). This potentially precedes the condition in male pupae, which appear to have lost the bigger structures accommodating the symbionts (Figure 4F). In addition, we observed that the symbiotic organs in larvae looked almost symmetrical in sagittal sections (Figure 4B), whereas the caudal side of the organ looked more pronounced in the female pupae (Figures 4C,D). Overall, these observations suggest that the symbiotic organs of males are possibly already detached shortly before pupation and are shed off with the last larval molt. By contrast, females retain their symbiotic organs during the last larval molt but undergo morphological changes, leading to one enlarged first organ with the majority of the symbionts, and two remnants of the former second and third organ.

### Symbiont transmission from pupa to female adults

**FIGURE 5:**
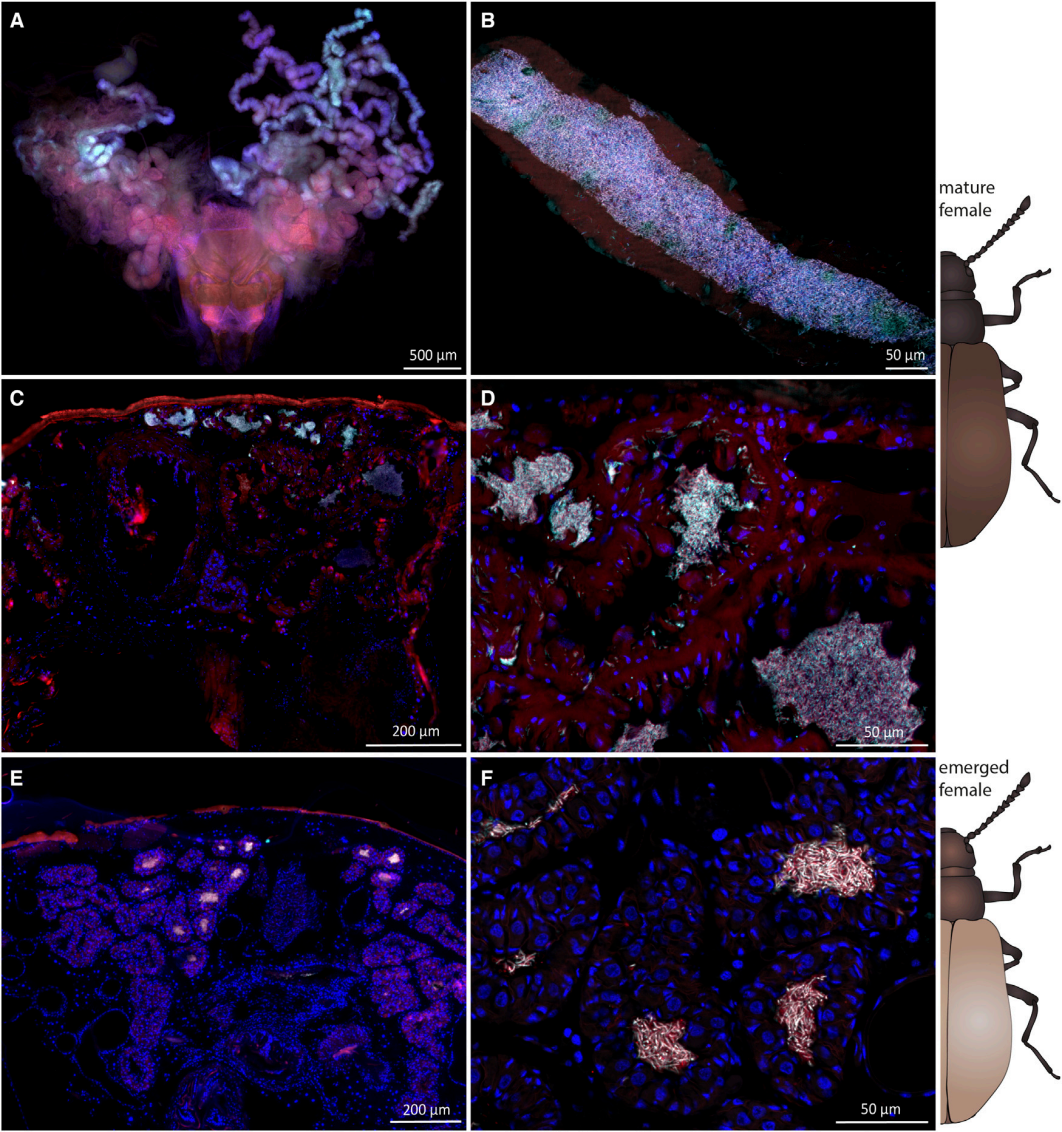
Symbiont presence and localization in mature and recently emerged *L. villosa* females. FISH images of the accessory glands of mature **(A–D)** and freshly emerged **(E,F)** field-collected females. Symbionts are generally depicted in white, host cuticle and tissue in red and host nuclei in blue. **(A)** FISH of a whole-mount dissected ovipositor and accessory glands showing the tubular structure of the symbiont-bearing organ. **(B)** Higher magnification of one tube which is densely filled with bacteria. **(C,D)** Transversal section through a mature female abdomen showing symbionts within the accessory glands. **(E,F)** Transversal section through a freshly emerged adult also showing dense accumulations of symbionts within the accessory glands. For **(A–D)** *Burkholderia-specific* staining is shown in red, Lv-StB specific staining in cyan, and host cell nuclei in blue (DAPI). For **(E–F)** Lv-StB specific staining is shown in cyan, general eubacterial staining in red, and host cell nuclei in blue (DAPI). Overlapping signal is shown in white.

**FIGURE 6:**
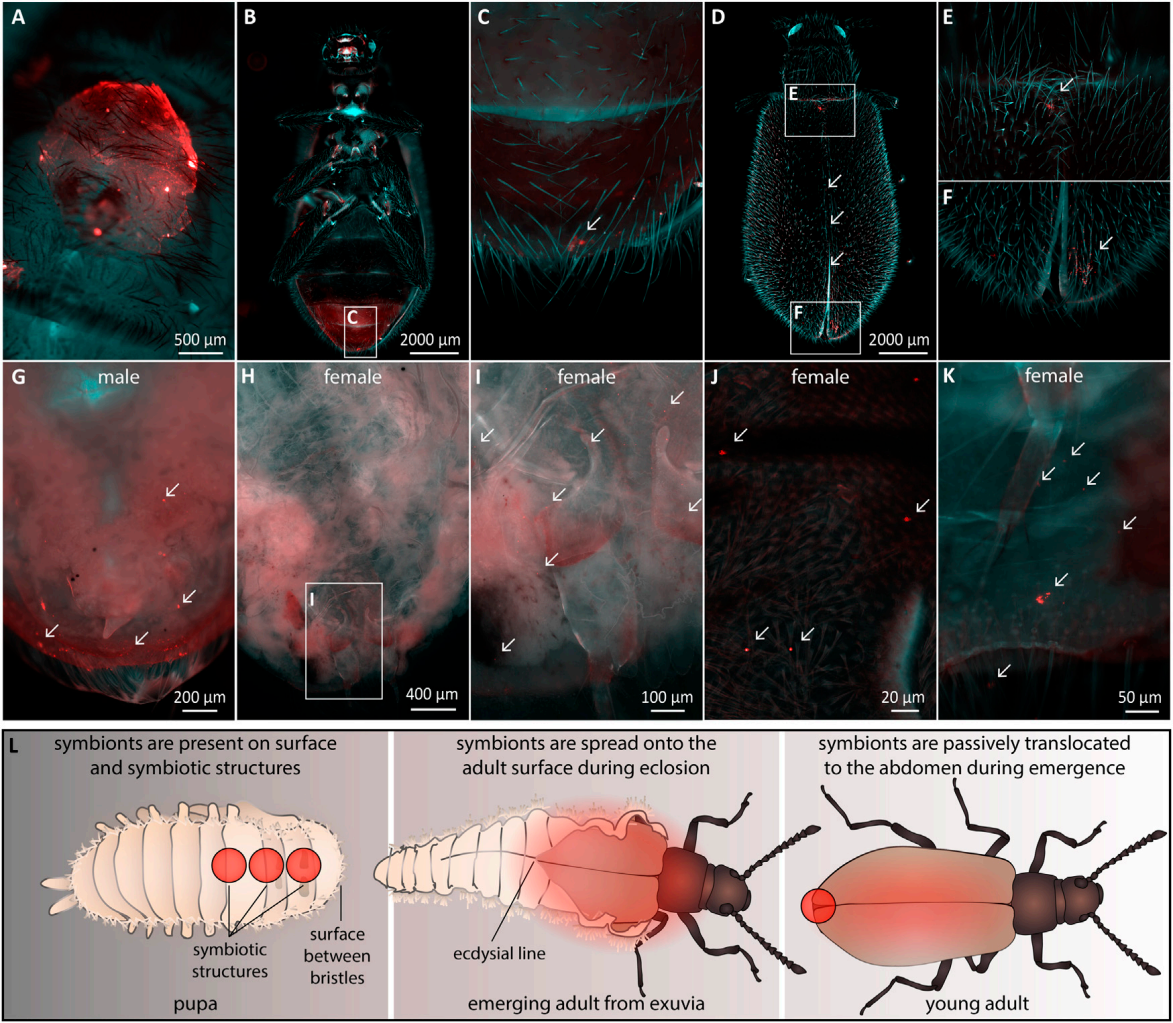
Transmission of immotile beads from the dorsal thorax of pupae to the abdominal region in adults. Fluorescent beads were applied to the region of the dorsal symbiont organ in *L. villosa* pupae and their location was examined on emerged adults. Fluorescent beads (arrows) are shown in red. The general autofluorescence of the insects in the EGFP channel is shown in cyan, while weak autofluorescence was also present in red. **(A)** Location of the applied beads on the surface of a pupa. **(B)** Ventral view of an emerged adult showing beads in the caudal abdominal region **(C)**. **(D)** Dorsal view of an adult showing beads on the thorax and abdomen **(E)** and at the caudal part on the elytra **(F)**. **(G)** Dorsal view of a male abdomen, where elytra and integument were removed showing accumulations of beads. **(H–J)** Dorsal view of one female abdomen without elytra and integument, showing multiple beads in the region of the reproductive system. **(K)** Dissected abdomen of a second female showing several beads in the abdomen. **(L)** Proposed mechanism of the translocation of beads and symbionts during eclosion. Red circles or areas indicate the presence of symbionts or fluorescent beads observed across our experiments.

It is unclear how symbionts are transmitted from the dorsal structures in pupae to the accessory glands of the reproductive system in female *Lagria* beetles (Figures 5A,B). In our FISH experiments, we only detected symbionts on the outer surface (Supplementary Figure S2) and in the cuticular organs of pupae, but never inside the body. Therefore, we hypothesized a transfer of the symbionts on the body surface to the reproductive tissues during metamorphosis, as proposed for *L. hirta* and observed for the sucking louse *Haematopinus*, where symbionts are transmitted through the molting fluid towards their origin into the ovaries of females (Buchner, 1953). Hence, we investigated when and how the symbionts colonize the accessory glands of female *L. villosa*. We first compared accessory glands of a mature and a newly emerged female via FISH to evaluate potential differences in symbiont presence and quantity. In mature females, the accessory glands are densely packed with symbionts (Figures 5C,D), and dense symbiont cultures were also observed in the newly emerged female (Figures 5E,F). We estimate that this specimen had emerged within a few hours prior to fixation, as deduced from its light and incompletely melanized wings when fixated. Nonetheless, it housed a large number of symbionts, which suggests either transmission of many symbionts from pupa to adult, or a notably fast symbiont population growth rate within the accessory glands. Since the dominant symbiont strain in *L. villosa* beetles, *B. gladioli* Lv-StB does not have genes involved in chemotaxis or flagella assembly (Waterworth et al., 2020), we were interested in whether an immotile bacterium could be transmitted over a distance from the thorax to the abdomen. Therefore, we used fluorescent latex beads to simulate immotile symbionts of comparable size (1 µm) and tracked them during pupation via microscopy (Figure 6). We applied 10^6^ latex beads to the dorsal thorax of pupae resembling natural symbiont titers (Figure 6A) and then localized the beads in newly emerged adults. We found the beads at the caudal end of the ventral abdomen (Figures 6B,C) as well as on the elytra (Figure 6D) near the thorax (Figure 6E) and on the dorsal caudal end (Figure 6F). After removal of the elytra and integument, beads were found in the caudal region of a male beetle (Figure 6G) and in a female beetle in the region of the reproductive system (Figures 6H–K). These observations suggest that immotile symbionts can be externally translocated by the host from the dorsal thorax to the region around the reproductive system (Figure 6L). From there, they likely colonize the symbiotic organs only present in females by so far unknown mechanisms.

## Discussion

Here we characterize the developmental dynamics of the symbiosis between *Burkholderia* ectosymbionts and the beetle species *L. hirta* and *L. villosa,* specifically during the host pupal stage*.* By quantifying and localizing the symbionts, detailing the structure of the symbiotic organs, and simulating a possible transmission route for the symbionts during pupation, we gain novel insights into the morphological rearrangements and the role of specialized symbiotic organs during metamorphosis of this insect.

Morphological adaptations to accommodate microbial symbionts have evolved in many insect taxa. Sometimes, these adaptations are associated with the exoskeleton of the insect as modifications of the cuticle (Stammer, 1929; Buchner, 1953; Li et al., 2018; Biedermann and Vega, 2020; Goettler et al., 2022). Among those, the symbiotic organs in *Lagria* larvae and pupae are unique given their morphology and presence on the dorsal thorax. However, their developmental origin has not been elucidated yet. These are cuticle-lined invaginations associated with glandular cells (Stammer, 1929), which likely aid in nourishing the symbionts via secretions. Similarly, mycangia are structures accommodating mainly fungal symbionts in bark and ambrosia beetles (Kirkendall et al., 2015; Hulcr and Stelinski, 2017), lizard beetles (Toki et al., 2012), ship-timber beetles (Toki, 2021), stag beetles (Kubota et al., 2020), leaf-rolling weevils (Wang et al., 2015) and also wood wasps (Kajimura, 2000). In ambrosia beetles, large variation in anatomy and location of mycangia is described, while their purpose is generally symbiont cultivation and transmission (Biedermann and Vega, 2020). Glandular cells, likely for nourishing the symbionts, are associated with the mycangia (Mayers et al., 2020). Some mycangia occur in a similar region as the *Lagria* organs at the dorsal thorax (Mayers et al., 2020), but seem to be only present in adult beetles.

Unlike the nutritional benefit in mycangial symbioses in beetles, other cuticle-associated symbionts protect their hosts, food source, or offspring against pathogens. Adult attine ants harbor defensive *Pseudonocardia* symbionts within cuticular crypts (Currie et al., 2006; Li et al., 2018; Goldstein and Klassen, 2020), antibiotic-producing *Streptomyces* symbionts are located within antennal gland reservoirs and on the pupal cocoon of beewolves (Kaltenpoth et al., 2005; Goettler et al., 2007, 2022; Kroiss et al., 2010), and protective *Penicillium* fungi colonize mycangia of the leaf-rolling weevil *Euops chinensis* (Wang et al., 2015). In these systems, immature host stages lack specialized structures to accommodate their symbionts, which might be due to the sheltered lifestyle within burrows, galleries, chambers, or leaf cradles during development. In those habitats, there might be no selective pressure to evolve or retain specialized structures, if symbionts can be deposited along with the offspring and taken up later.

Storing the symbionts outside the host in the environment can be a way to circumvent problems associated with symbiont transmission during complete metamorphosis (Shukla et al., 2018; Hammer and Moran, 2019), which often requires symbiont translocations across morphologically different life stages. In eusocial insects, such as bees (Powell et al., 2014) or ants (Poulsen et al., 2003) with overlapping generations in a shared environment, social transmission allows the host to have aposymbiotic life stages without losing the association to symbionts (Onchuru et al., 2018). For solitary holometabolous hosts without this opportunity, maintaining beneficial symbiotic organs and symbionts can be problematic, especially during pupation, when the larval tissue is remodeled into the completely distinct adult stage (Hammer and Moran, 2019; Rolff et al., 2019). Symbionts that are located internally or associated with the gut epithelium can be especially affected by this reorganization. Degradation and remodeling of the gut might lead to elimination or shifts in microbial communities (Rolff et al., 2019; Manthey et al., 2021), however, a core microbiota can sometimes be maintained even under drastic conditions (Johnston and Rolff, 2015). *Lagria* pupae circumvent the risk of losing symbionts during metamorphosis through specialized structures that enable maintenance and direct transmission of symbionts during female development. In addition, their defensive symbionts stay connected with the host in every life stage. This might facilitate free foraging in the environment without constraints of building or finding specialized habitats for environmental symbiont transmission during delicate phases, like molting or pupation.

Although *Lagria* beetles continuously house *Burkholderia* symbionts, they offer different habitats during their life cycle. The conditions for the symbionts might change not only due to occasional horizontal transmission through the plant environment (Wierz et al., 2021) but also within the beetle. Starting from direct exposure to the environment on the egg stage for around 6 days, they colonize a more confined habitat with contact to the environment in the dorsal structures of larvae. Then, they inhabit an again more exposed stage on the surface during pupation, which usually lasts 6 days, until they reach their final and probably most sheltered condition in the female accessory glands. Changes in the environment, even if minor, impose selective pressures on the symbionts and might force them to adapt to different abiotic and biotic factors, e.g., temperature, pH, nutrients, or host immune factors (Cao and Goodrich-Blair, 2017). While we cannot draw conclusions on the molecular factors that are involved in symbiont colonization and relocation, we can suggest potential mechanisms for translocation of the bacteria based on our results on the morphology and development of the symbiotic organs across life stages. It is plausible that motile members of *Lagria* beetle microbiota can actively move longer distances during translocation to another organ, yet Lv-StB (Waterworth et al., 2020) can colonize despite being presumably immotile. Here, we show that accessory glands of early emerged adult females are already highly infected with the symbionts, including Lv-StB. Therefore, it is possible that colonization by a few cells is sufficient, and they can rapidly grow within the glands in the first hours after emergence. However, the precise growth rate of Lv-StB during this period has not been determined.

Because we have not detected symbionts within the guts of female pupae and never within host cells, an internal transmission to the reproductive system during pupation seems unlikely (Buchner, 1953). The transfer of immotile beads into the region of the symbiotic organs suggests that host movements might contribute to translocation of symbionts without fully relying on bacterial motility (Figure 6L). However, the relevance of motility to finally colonize the structures remains uncertain. In many vertically transmitted symbionts, relaxed selective pressures on genes involved in motility and chemotaxis have led to the loss of these genes (Moran et al., 2008). For intracellular symbionts, motility is not needed for transmission when symbionts are consistently present in germ line cells (Serbus et al., 2008) or they can rely on host-mediated transport mechanisms (Koga et al., 2012). Endosymbionts that are only located in bacteriomes can also be transmitted via migrating bacteriocytes (Luan et al., 2016; Maire et al., 2020). Also, many pathogens are non-motile, yet able to infect hosts (Matilla and Krell, 2018), often by being transported via hitchhiking on other bacteria (Muok and Briegel, 2021). For gut symbionts, host behaviors such as peristaltic movements might also help symbionts reach their niche after they are ingested orally, making motility expendable (Bright and Bulgheresi, 2010). Migration via the molting fluid, i.e., the liquid accumulating between old and new cuticles before each molt, is also possible and could exempt symbionts of specialized motility. This has been described in bacterial symbionts of *Haematopinus* lice, harbored in three symbiotic “depots” that develop in the dorsal area of female larvae, close to the midgut (Buchner, 1953). Before the final molt leading to adulthood, the bacteria are released from these depots into the molting fluid, and thereby reach an opening to the developing reproductive organs. This allows the symbionts to colonize ovarial ampullae and later facilitate vertical transmission to the eggs (Ries, 1931; Buchner, 1953). While apparently similar, the dorsal depots of lice have a different histological origin to the symbiotic structures of *Lagria* larvae, as they are not cuticular. Also, based on our histological observations, the dorsal crypts of *L. villosa* are unlikely to have access to the molting fluid (Figures 4B–F). Instead, immotile *Lagria* symbionts are possibly translocated passively during emergence from the exuvia. Exuviae open at the cranial end of the dorsal ecdysial line exactly at the location of the symbiotic organs, from where the freshly emerged adults crawl out. This breaking point might facilitate symbiont transmission, ensuring that the host is infected with the symbionts from the exuvial surface during emergence (Figure 6L). However, it is possible that some kind of motility is necessary to finally reach the accessory glands in adult females.

The defensive function of *Burkholderia* symbionts was previously described via manipulative bioassays for *L. villosa* eggs (Flórez et al., 2017, 2018) and young larvae (Janke et al., in review). A defensive role of the symbionts during the pupal stage is also possible, since bioactive compounds could be detected in organs of *L. villosa* pupae and the respective exuviae (Janke et al., in review). However, since our current results show that *L. villosa* and *L. hirta* female pupae contain higher symbiont titers than the male counterparts, the latter may be less well defended. While low titers of symbionts in addition to remnants of the defensive metabolites may be sufficient for aiding in protection during male pupation, this requires further investigation. Plausibly, transmission to the female accessory glands might be the major selective pressure to maintain symbionts during pupation.

Although metamorphosis is a key driver of adaptability and diversity in holometabola, it comes with the constraints of higher vulnerability during pupation and the need to relocate beneficial symbionts (Hammer and Moran, 2019; Rolff et al., 2019). While numerous taxa within most holometabolous insect orders are described to harbor mutualistic microbes, their maintenance and relocation in immature life stages (i.e., larvae and pupae) and specifically during metamorphosis are only rarely studied. Here, we elucidate the morphology of peculiar symbiotic structures of the exoskeleton in pupae of two *Lagria* species and propose the transmission route of ectosymbionts during metamorphosis. The morphological modification of the dorsal cuticle allows the beetle to retain the valuable symbionts in a reservoir despite remodeling of internal structures during metamorphosis.

## Data Availability

The original contributions presented in the study are included in the article/Supplementary Material, further inquiries can be directed to the corresponding author.
