# Supplementary material for: Morphological adaptation for ectosymbiont maintenance and transmission during metamorphosis in Lagria beetles
Source: Front Physiol. 2022 Aug 30;13:979200. doi: 10.3389/fphys.2022.979200 (PMC9468232; doi:10.3389/fphys.2022.979200)
Supplement: Supplementary file 3 [file DataSheet1.docx]

Supplementary Material

# Supplementary Data

Video S1: µCT through a female *L. villosa* pupa. Symbiotic structures visualized in red.

Video S2: µCT through a male *L. villosa* pupa. Symbiotic structures visualized in red.

# Supplementary Figures and Tables

## Supplementary Figures


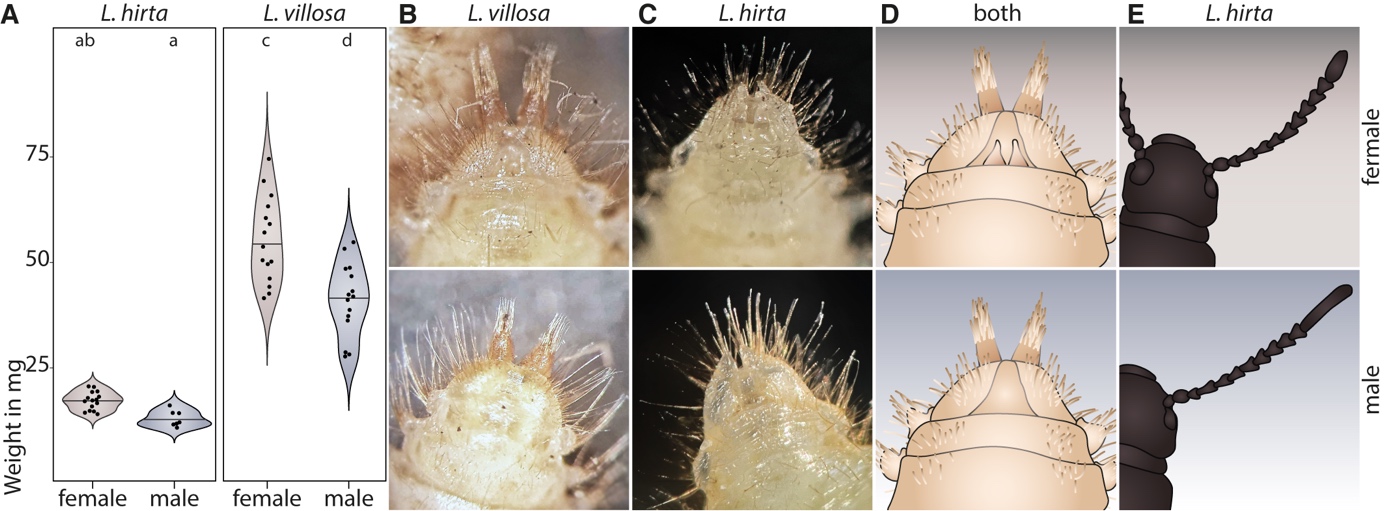


**Supplementary Figure 1.** Sex determination of *Lagria* pupae. (A) Weight of early *Lagria* pupae. (B-E) Morphological characteristics of female (upper row) and male (lower row) pupae. (B, C) Photographs of the ventral side of the caudal region of the abdomen of female and male *L. villosa* (B) and *L. hirta* (C) pupae. (D) Illustration showing visual differences between female and male pupae. E) Differences on the length of the last antennal segment of *L. hirta* beetles, which can be observed in the pupal and adult stage.

**
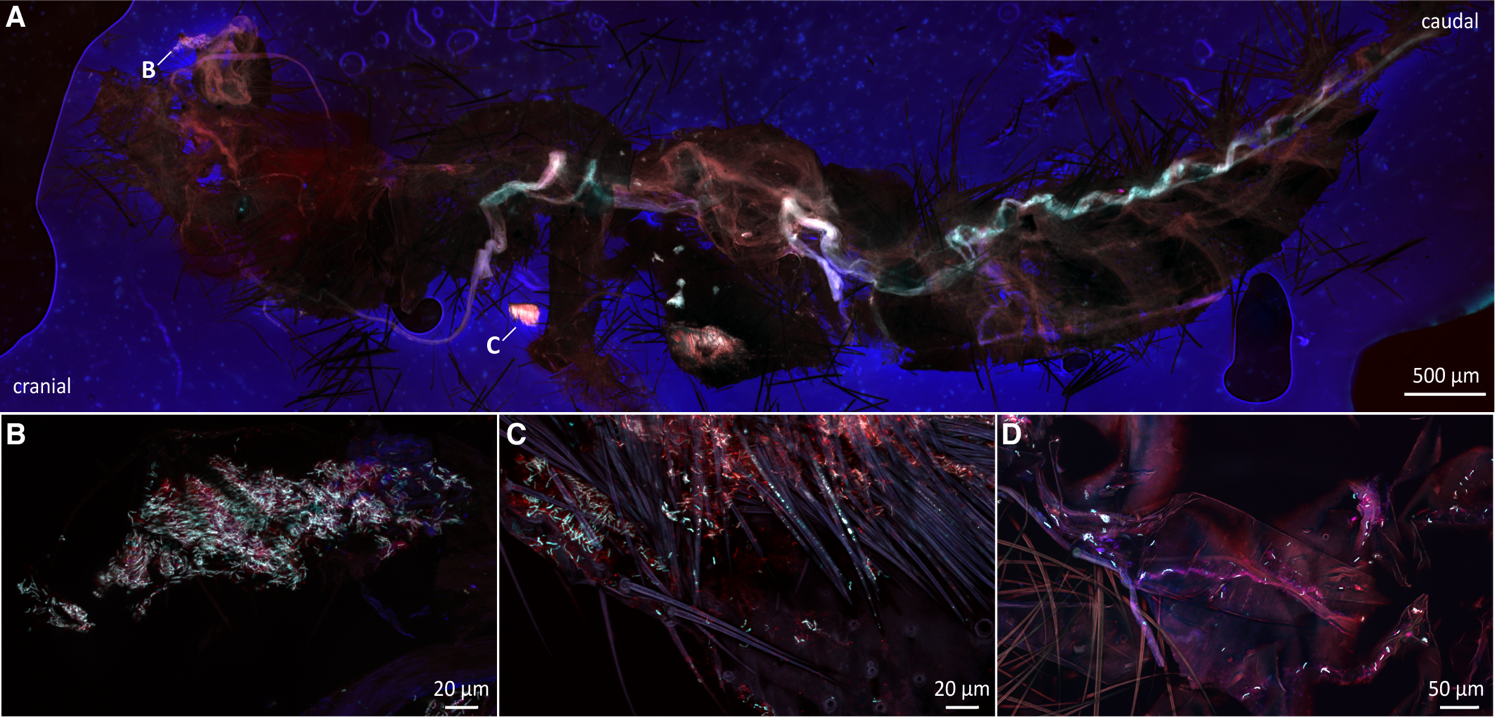
**

**Supplementary Figure 2.** Symbiont localization on the surface of *L. villosa* pupae. Symbionts are generally depicted in white, host cuticle and tissue in purple and the adhesive tape in blue. *Burkholderia-specific* staining is shown in cyan, eubacterial specific staining in red, and overlapping cells in white. (A) FISH on an adult-pupa exuvia collected from the field. (B, C) Dense symbiont accumulations in different parts of the exuvia shown in (A), including the external surface, as evidenced by the insect bristles (C). (D) Symbionts spread out across the exuvia.

Supplementary Table 1: Original localities of collected beetles and their use for this study

| Time | State | Locality | Coordinates | Habitat | Species |
| --- | --- | --- | --- | --- | --- |
| July,  2020 | Rheinland-Pfalz, Germany | Höhr-Grenzhausen | 50°26'12.8"N,  7°40'46.7"E | Mixed coniferous forest | *L. hirta* |
| July,  2020 | Rheinland-Pfalz, Germany | Höhr-Grenzhausen | 50°26'26.4"N,  7°40'50.2"E | Mixed coniferous forest | *L. hirta* |
| May 2019 | São Paulo, Brazil | Jundiaí | S23° 8' 3.732",  W46° 58' 47.352" | Manioc plantation | *L. villosa* |
| May 2019 | São Paulo, Brazil | Jundiaí | S23° 7' 42.06",  W46° 59' 25.296" | Starfruit tree | *L. villosa* |
| May 2019 | São Paulo, Brazil | Cordeirópolis | S22° 30' 11.376",  W47° 25' 40.08" | Sugar cane plantation | *L. villosa* |
| May 2019 | São Paulo, Brazil | Cordeirópolis | S22° 30' 13.32",  W47° 25' 28.092" | Soybean, radish plantation | *L. villosa* |
| May 2019 | São Paulo, Brazil | Itirapina | S22° 15' 15.84",  W47° 50' 43.728" | Corn, coffee plantation | *L. villosa* |
| May 2019 | São Paulo, Brazil | Brotas | S22° 16' 18.516",  W47° 56' 4.452" | Manioc plantation | *L. villosa* |
| May 2019 | São Paulo, Brazil | Brotas | S22° 17' 25.98",  W48° 3' 12.276" | Rye plantation | *L. villosa* |
| March 2019 | São Paulo, Brazil | Cordeirópolis | S22° 29' 26.88",  W47° 25' 58.476" | Soybean plantation | *L. villosa* |
| March 2019 | São Paulo, Brazil | Brotas | S22° 17' 25.98",  W48° 3' 12.276" | Soybean plantation | *L. villosa* |
| March 2019 | São Paulo, Brazil | Jaú | S22° 15' 49.896",  W48° 31' 12.396" | Soybean plantation | *L. villosa* |
| March 2019 | São Paulo, Brazil | Cordeirópolis | S22° 29' 40.776",  W47° 23' 48.192" | Soybean plantation | *L. villosa* |
| March 2019 | São Paulo, Brazil | Santa Gertrudes | S22° 27' 56.196",  W47° 31' 55.488" | Soybean plantation | *L. villosa* |
| March 2019 | São Paulo, Brazil | Cordeirópolis | S22° 30' 11.376",  W47° 25' 40.08" | Soybean plantation | *L. villosa* |
| March 2022 | São Paulo, Brazil | Cordeirópolis | S22°30'11.4"  W47°25'40.1" | Soybean plantation | *L. villosa* |
| March 2022 | São Paulo, Brazil | Cordeirópolis | S22°29'27.1" W47°26'04.7" | Soybean plantation | *L. villosa* |
| March 2022 | São Paulo, Brazil | Cordeirópolis | S22°29'13.1" W47°26'34.8" | Soybean plantation | *L. villosa* |
| March 2022 | São Paulo, Brazil | Cordeirópolis | S22°28'42.8" W47°26'38.0" | Soybean plantation | *L. villosa* |
| March 2022 | São Paulo, Brazil | Torrinha | S22°23'41.4" W48°08'43.1" | Soybean plantation | *L. villosa* |
